# The Enigmatic Origin of Papillomavirus Protein Domains

**DOI:** 10.3390/v9090240

**Published:** 2017-08-23

**Authors:** Mikk Puustusmaa, Heleri Kirsip, Kevin Gaston, Aare Abroi

**Affiliations:** 1 Department of Bioinformatics, University of Tartu, Riia 23a, Tartu 51010, Estonia; mikk.puustusmaa@ut.ee (M.P.); heleri16@ut.ee (H.K.); 2 School of Biochemistry, University of Bristol, Bristol BS8 1TD, UK; kevin.gaston@bristol.ac.uk; 3 Estonian Biocentre, Riia 23b, Tartu 51010, Estonia; 4 Institute of Technology, University of Tartu, Nooruse 1, Tartu 50411, Estonia

**Keywords:** papillomaviruses, protein domains, structural domains, origin

## Abstract

Almost a century has passed since the discovery of papillomaviruses. A few decades of research have given a wealth of information on the molecular biology of papillomaviruses. Several excellent studies have been performed looking at the long- and short-term evolution of these viruses. However, when and how papillomaviruses originate is still a mystery. In this study, we systematically searched the (sequenced) biosphere to find distant homologs of papillomaviral protein domains. Our data show that, even including structural information, which allows us to find deeper evolutionary relationships compared to sequence-only based methods, only half of the protein domains in papillomaviruses have relatives in the rest of the biosphere. We show that the major capsid protein L1 and the replication protein E1 have relatives in several viral families, sharing three protein domains with *Polyomaviridae* and *Parvoviridae*. However, only the E1 replication protein has connections with cellular organisms. Most likely, the papillomavirus ancestor is of marine origin, a biotope that is not very well sequenced at the present time. Nevertheless, there is no evidence as to how papillomaviruses originated and how they became vertebrate and epithelium specific.

## 1. Introduction

Members of the *Papillomaviridae* taxonomic family have a small circular double-stranded DNA genome of around 8kb in length that is packaged in a non-enveloped icosahedral capsid. Papillomaviruses (PVs) have been particularly well-studied in humans due to their association with multiple disease states including cervical cancer and other malignancies. Well over 200 human papillomavirus (HPV) types have been identified to date. Historically, the first discovered PV was Cottontail rabbit PV (current name SfPV1), which was also the first DNA tumour virus described [1]. PVs infect most mammal species (both terrestrial and marine), several birds, reptiles, and fish [2,3]. In well-studied host species, some PV type infections are asymptomatic; therefore, in-depth study of vertebrates’ epithelial viromes may significantly increase the number of known PVs. After the first fully sequenced PV genomes were published [4,5], the first sequence analyses of PVs were also performed [6,7,8,9,10]. Subsequently, there have been several studies of the ancestral and more recent evolution of PVs [11,12,13,14,15]. However, the evolutionary origin of PVs is not well understood, although it is assumed to be ancient.

PV sequences can be found in different nucleotide databases: in ENA (European Nucleotide Archive) there are ~25,000 sequences, and in NCBI (National Center for Biotechnology Information) there are 25,189 sequences with the taxonomic restriction *Papillomaviridae*. In NCBI 1686 entries are found with length 6300 to 9500 nucleotides and with taxonomic restriction *Papillomaviridae*, mostly corresponding to PV complete genomes (this redundant set includes isolates, etc.). “NCBI refseq”, which is a subset of the NCBI nucleotide collection containing only reference genomes (a non-redundant database), contains 135 reference PV genomes. In the UniProtKB (UniProt Knowledgebase) database, there are 556 entries in the manually annotated SwissProt and 12,302 in the computer-annotated TrEMBL (TrEMBL contains the translations of all coding sequences present in the EMBL Nucleotide Sequence Database not yet integrated in Swiss-Prot). In UniProt “complete proteomes” (“complete proteome”—all proteins annotated for species or isolate), 97 PV proteomes can be found, including 37 “reference proteomes” (“reference proteomes” are a representative cross-section of the taxonomic diversity to be found within UniProtKB “complete proteome”, they include the proteomes of well-studied model organisms and other proteomes of interest for biomedical and biotechnological research; for more details, see [16,17]. In the PAVE (Papillomavirus Episteme [2]) database, which was curated by experts in the field, 340 PV types with 3150 protein sequences are found (as of 8 June 2017) [3]. However, whether sequence information alone is enough to tell us something about the deep evolutionary history of PVs and their origin is open to debate.

Viruses are fast evolving units. PV coding sequences have been estimated to evolve ~5 times faster on average compared to their mammalian host nuclear coding sequences [14]. The evolutionary rate of the PV E1 protein is estimated to be 1.76 × 10^−8^ substitutions/nt/year for Lambdapapillomaviruses infecting Felidae; 7.1 × 10^−9^ substitutions/nt/year for mammalian PVs; and 1.1 × 10^−8^ substitutions/nt/year for nonmammalian amniote PVs [11,18,19,20] compared to 2.2 × 10^−9^ for mammalian nuclear coding sequences [21]. In general, the short-term evolutionary rates of viruses (and other genomes) are much faster than long-term evolutionary rates due in part at least to the loss of deleterious mutations from the population [22]. Thus, the sequence space sampled by viruses is even larger than that expected from long-term evolutionary rates. It is estimated that PVs have existed at least ~315 million years [23]. Considering this, PV proteins may still have homologs in the biosphere (outside of PVs), but without significant sequence similarity.

It has been known for more than three decades that structure is more conserved than sequence [24,25]. Challis and Schmidler have shown that including structural information enables better phylogenetic inference for distant relationships [26]. Additionally, Herman et al. have shown that including structural information reduces significantly the uncertainty of alignments and topologies of phylogenetic trees, indicating that structure contains more information than can be obtained from sequences alone [27]. This is especially important in the case of viruses, which are able to sample a huge amount of sequence space and loose sequence similarity within a relatively short time (compared to organisms). Thus, it is essential to include structural information in order to study deep evolutionary relationships.

A common view of proteins is that they are composed of domains—independent functional, evolutionary and structural units often linked by unstructured polypeptide chain. A protein (polypeptide chain) can be virtually chopped into domains on multiple criteria and domain borders depend on the domain assignment method. Domains are more monophyletic compared to proteins as one protein may consist of many domains with very different phylogenetic histories. Thus, protein domains, and especially structural domains, can be used to study the evolutionary history (origin) of viral proteins.

In this study, the structural information of protein domains was used to find distant homologs to PV proteins and to shed more light on the evolutionary history of PVs. Our results show that only half of the PV protein domains have a relative in the rest of the sequenced biosphere. E1 replication protein shows the most connections with cellular organisms and viruses alike. Capsid protein L1 has evolutionary relationship with rest of the virosphere. However, for a number of PV protein domains, distant homologs could not be detected.

## 2. Materials and Methods

### 2.1. PfamA_28

In this study (if not mentioned otherwise), locally downloaded version of PfamA_28 (based on Swiss 2014_07 + SP-TrEMBL 2014_07) was used instead of the newest version of PfamA for reasons described in the Supplementary Data [28].

Protein domain models in PfamA, and also in SUPERFAMILY [29], are based on profile Hidden Markov models (profile-HMMs), which are widely used for modelling protein or nucleotide consensus sequence. A profile-HMM is constructed from a multiple sequence alignment, which is called the seed alignment, containing a set of representative members of the protein domain family. A query sequence that has a significant score against the profile-HMM is considered homologous to the (seed) sequences that were used to build the profile-HMM. In PfamA, the whole protein domain (PfamA entry) is described by a single HMM. PfamA_28 contains a diverse collection of protein domain families mapped to all available UniProt sequences.

By default, the non-redundant “complete proteomes” subset of UniProt is used here because of the quality of the data and because the coverage of the data can be confidently interpreted. Full UniProt is highly redundant and biased, which makes interpretation of coverage of the data questionable. However, to broaden the scope of our analyses and to evaluate the occurrence of PV_PfamA protein domains in non-complete proteomes, the full Uniprot was used.

### 2.2. HMMER “Hmmsearch”

PfamA_28 is based on the sequence data from summer 2014 (UniProt version 2014_07). To look for the occurrence of PV protein domains in recently added sequences in databases, we used HMMER web tool [30] to perform “hmmsearch” (searching protein alignment/profile-HMM from protein sequence database) against UniProt “complete proteome”, full UniProt and Ensemble databases with PfamA and SUPERFAMILY profile-HMM models listed in Supplementary Table S2 as queries [31]. “Hmmsearch” was performed in March/April 2017 with default settings.

### 2.3. Criteria for Considering PfamA_28 Database Hits and “Hmmsearch” Hits as True Positives

In PfamA, which is based on high throughput data, every specific case needs to be analysed in detail to avoid including false positives and making premature conclusions. We applied the following additional criteria to PfamA_28 database hits and to HMMER hits (with PfamA models) before considering them as true positives (and to exclude them as false positives if not satisfied):

1. Sequence annotation is valid (not showing evidence for viral contamination);
2. The size and protein coding potential of the cellular contig/scaffold should exclude the possibility of viral contamination by small viruses (applied to complete genome/proteomes);
3. “hmmscan” (protein sequence vs. profile-HMM database with HMMER) gives reciprocal best hit to query PfamA model; and
4. 3D structure prediction by threading meta server LOMETS gives best modelling templates from PV structures at least with one algorithm [32].

Protein 3D structure prediction has been used before to validate sequence based hits of non-vertebrate polyomaviruses [33]. LOMETS meta server is based on multiple primary algorithms predicting 3D structure (algorithms listed in Supplementary Data) [32]. A criterion for true positives was applied when a number of hits in superkingdom or in viruses did not exceed 50 species.

### 2.4. Galaxy of Folds

Location of PV structural domains in global structure space was visualised in the “galaxy of folds”, which is based on the sequence similarity of a non-redundant set of SCOP domains [34]. SCOP database (the Structural Classification of Proteins) is a classification of protein structural domains (SCOP domains) based on similarities of their structures and amino acid sequences [35]. Alva et al. conducted an all-against-all comparison of SCOP domains with <20% pairwise identity. Domains were clustered using a force-directed procedure, and the statistical significance of pairwise comparisons was used to assign attractive and repulsive forces to each profile pair in a two-dimensional map [34]. Because of the force directed clustering procedure, domains find their equilibrium position on the map not only by attraction to similar domains but also by repulsion of different ones. “Galaxy of folds” visualisation tool was used to map PV domains to the structural space [34,36].

### 2.5. SUPERFAMILY Database

SUPERFAMILY database was locally downloaded (October 2014) and based on SCOP 1.75 [29,37]. Protein domain (and domain pair) existence in PVs and their distribution in Archaea, Bacteria, and Eukaryota were obtained from the “len_supra” table. Option include = “y” was used in queries against cellular complete genomes, to remove isolates, strains, etc. Information about PV_SF distribution in viruses and plasmids was obtained from “sublen_supra” table, option genome = “vl” or genome = “pla” was used respectively. To extend the queries to non-complete genomes, “sublen_supra” table was used with option genome = “up” and with respective taxonomic restriction.

### 2.6. Criteria for Considering Hits from SUPERFAMILY Database and from “Hmmsearch” as True Positives

As in PfamA data, we applied criteria to avoid false positives. Similar criteria to PfamA data were used:

(1) Sequence annotation is correct (for UniProt data);
(2) The size and protein coding potential of the cellular contig/scaffold exclude viral contamination by small viruses (applied to complete genomes);
(3) “hmmscan” gives reciprocal best hit to query SF model; and
(4) 3D structure prediction by threading meta server gives best modelling templates from respective SF at least with one algorithm.

For true positive eukaryotic hits in UniProt sequences, annotations of corresponding nucleic acid sequences (as provided by UniProt homepage) were examined to find more information about the origin of the sequences (coded by eukaryotic mitochondria, eukaryotic plasmids, etc.). Criteria for true positives were applied when a number of hits in superkingdom or in viruses did not exceed 50 species.

## 3. Results

### 3.1. PfamA Protein Domains Found in PV

PfamA is one widely used protein domain database. As a first approximation, PfamA is sequence and function based. According to the PfamA_28 database, 12 PfamA domains are found in PVs (collectively named PV_PfamA). On average, about 90% of proteins in PVs are covered by at least one PfamA domain (Table 1). In addition, about 84% of amino acids in PVs are covered by PfamA domains (Table 1). Compared to cellular superkingdoms and double-stranded DNA (dsDNA) viruses, PVs are very well covered with PfamA domains (Table 1).

**Figure 1:**
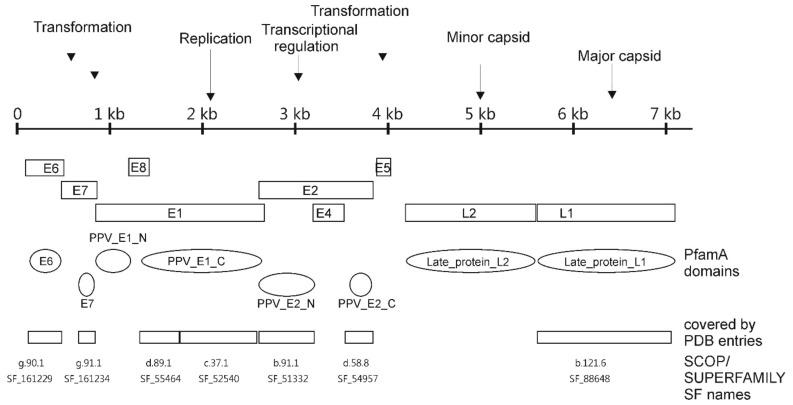
Location of Papillomavirus (PV) proteins and protein domains using Bovine PV type 1 as an example. Bovine PV type 1 encodes 9 proteins including the oncoproteins E6, E7 and E5, the viral helicase E1, the helicase loading factor and transcription factor E2, and the L1 and L2 coat proteins. E8^E2 and E1^E4 proteins are not shown on the figure. Location of open reading frames (ORFs) does not correspond to reading frames.

Excluding short N- and C-terminal regions, only two regions internal to the PV proteins are not assigned to PfamA domains. The short region between “PPV_E1_N” and “PPV_E1_C”, and the E2 “hinge region” (Figure 1). However, the E2 “hinge region” also encodes the E4 part of the E1^E4 protein, although in another reading frame with respect to E2. In UniProt “complete proteomes” the E4 open reading frame (ORF) (and E1^E4 protein) is not annotated at all in many PV genomes. Additionally, in several UniProt “complete proteomes” (and in UniProt), many non-canonical PV ORFs are annotated, but not yet experimentally characterised, hence they might be misannotations. The potentially misannotated proteins reduce the “the percentage of coverage”. However, moving from the redundant set of tens of thousands of sequences to protein domains, we end up with less than 20 evolutionary units (protein domains).

### 3.2. Relationships of PVs to the Sequenced Biosphere According to PfamA Domains

Domains are more monophyletic compared to proteins, as one protein may consist of many domains with very different phylogenetic histories. Thus, the protein domains can be used to study the evolutionary history of viral proteins. In the PfamA database, only a single true positive hit (see Materials and Methods) for the PPV_E1_C domain is found (in PfamA “complete genomes”) (Table 2). PPV_E1_N gives only two true positive hits in PfamA “complete genomes” (Table 2). PPV_E1_N was found in two *Nosema* species among Fungi proteins: C4V8V5_NOSCE and R0MJR2_NOSB1 (Supplementary Materials, File S1). However, as no structure for PPV_E1_N is available, we cannot confirm homology via predicted structure similarity (see Material and Methods). It should be noted that this region in PV E1 sequences is not very well conserved and has low complexity. PPV_E1_C gives one hit to Bacteria (Table 2, Supplementary Materials, File S1), namely *Dickeya dadantii* protein E0SH87_DICD3. None of the PV_PfamA domains are found in any viruses outside *Papillomaviridae* in the database used (UniProt “complete proteomes”).

The PfamA_28 “complete proteomes” contains 76 PV proteomes. However, the “PPV_E1_C” domain was not found in two PV complete proteomes (HPV53 and HPV56; E1 protein is not annotated for these PV types) and “PPV_E1_N” was not found in 4 complete proteomes (*Fringilla coelebs* papillomavirus (isolate Chaffinch/Netherlands/Dutch), *Psittacus erithacus timneh* papillomavirus (isolate African grey parrot), HPV53, and HPV56). Detailed examination of DNA sequences for HPV53 and HPV56 clearly shows that the absence of “PPV_E1_C” domain is caused by misannotations. For HPV53 and HPV56 the reference genome/proteome is based on the first published sequence and in both types the first sequence has missing nucleotides in the E1 coding region. Most if not all isolates of HPV53 and HPV56 have annotated full-length E1 protein. Thus, misannotations are one reason why PAVE-like activities are important.

To extend the search to non-complete genomes of organisms and viruses, we looked at the presence of PV_PfamA domains in the full UniProt (excluding PVs) in PfamA database. It is expected that UniProt contains more misannotations and partial sequences compared to “complete proteomes”. Therefore, more false positives should be expected. The amount of all false positive hits can be seen in Table S1. We performed the analysis and tested for false positives as described in Materials and Methods. In general, the results were similar to “complete genomes” set—only PV_PfamA domains from the E1 protein gave significant hits. PPV_E1_C gave 20 hits to Bacteria, mostly from *Enterobacteriaceae* (Table 2, for more information of positive hits, including species name and full taxonomy see Supplementary Materials, File S1). PPV_E1_N gives hits to four eukaryotes: 3 *Nosema* species (Fungi) (including two species/protein from “complete genome” and protein T0L8A9_9MICR) and one Spermatophyta (Viridiplantae) (protein V7BKU5_PHAVU).

PfamA_28 is based on UniProt release 2014_07, therefore to acquire more up to date data, “hmmsearch” was used with PfamA HMMs listed in Table 2 as queries (HMM version numbers listed in Table S2). After thorough analysis of all hits, only one positive bacterial hit (Planctomycetaceae bacterium SCGC AG-212-D15, protein A0A177Q2P3_9PLAN) and one viral hit to Planaria asexual element, protein Q91S73_9VIRU for PPV_E1_C remained (Table 2, Supplementary Materials, File S1). No other PV_PfamA model gave a true positive hit to cellular sequences.

In “full UniProt” viruses, PPV_E1_C gives highly significant matches to *Polyomaviridae* Large-T and *Parvoviridae* NS1 proteins. This similarity has been observed previously, mostly based on shared common helicase motifs [38]. The reasons why sequences of *Polyomaviridae* and *Parvoviridae* have the best score for PPV_E1_C HMM are described in Supplementary Materials. However, a phylogenetic tree clearly separates *Polyomaviridae*, *Parvoviridae* and *Papillomaviridae* replication protein sequences into three distinct protein families (data not shown). With the exception of the E1 domains described above, other PV_PfamA HMM models did not give any true positive hits to proteins in viruses outside PV sequences.

### 3.3. Location of PV Domains in the “Galaxy of Folds”

**Figure 2:**
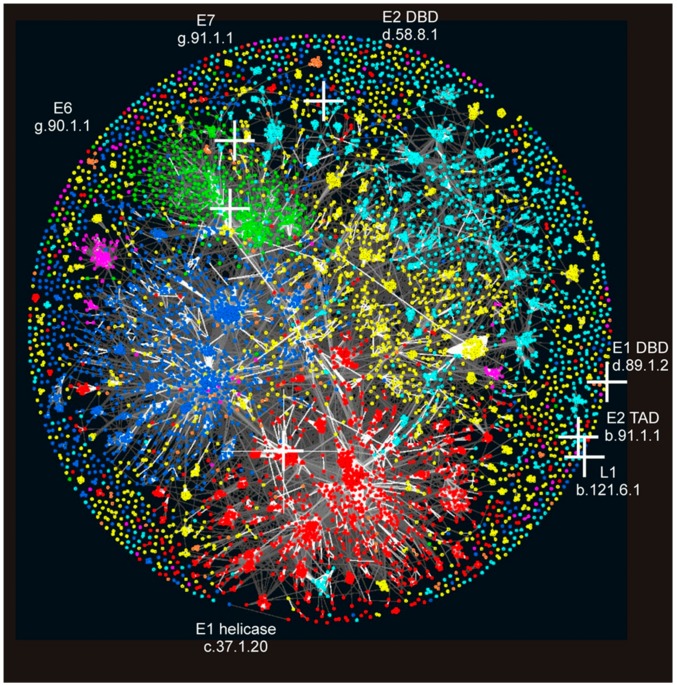
Location of PV domains in the “Galaxy of folds”. PV structural domains are marked by white crosses and visualised on protein domain space. Domains in Structural Classification of Proteins (SCOP) were clustered using the software CLANS based on their all-against-all pairwise similarities, as measured by HHsearch *p*-values [34]. Domains are coloured according to their SCOP class: all-a (blue); all-b (cyan); a/b (red); a + b (yellow), small proteins (green); multi-domain proteins (orange); and membrane proteins (magenta). PV protein domain name and SCOP identifier are indicated.

Occurrence of PV_PfamA domains in sequenced biosphere showed only weak connections with cellular organisms and other viruses. Therefore, structural information was included in our analysis. All PfamA domains found in PVs having a structural representative in Protein Data Bank (PDB) [39], are almost completely covered by longest PDB chain sequence, except E7, which is covered by about 50% (Table 2 and Figure 1). Overall, PVs are structurally very well characterised, especially among dsDNA viruses (see Supplementary in Reference [40]). Protein sequences in UniProt (or in other) databases can be chopped into domains on multiple criteria. Protein chains in PDB entries can be divided into domains according to criteria obtained from their 3D structure. As an example, this is done by hierarchical classification of protein domains in Structural Classification of Proteins (SCOP) and CATH databases [35,41]. In this work, SCOP database was used because it is more suitable for evolutionary studies. In addition to sequence similarity, SCOP protein domains are grouped together according to their structural similarity, according to the packaging of the core of the protein domain. SCOP has different hierarchical levels and one of them is Superfamily (SF) level. According to the SCOP authors, SF level is the highest level with confident homologous relationships. In PV protein structures, the SCOP domains cover most of the PDB chain (Figure 1, Table 2). However, this is not always the case. As noted earlier, proteins can be virtually chopped into domains on multiple criteria and domain borders depend on the assignment method. In PVs, there is good agreement (accordance) between the PfamA and SCOP domains (Figure 1). Only PfamA “PPV_E1_C” is separated into two domains in SCOP where E1 DNA-binding domain (DBD) forms a separate domain from E1 helicase domain (the latter includes a hexamerisation subdomain). In *Polyomaviridae*, Large-T protein and *Parvoviridae* NS1 protein the DBD and helicase domains are classified as separate domains in both PfamA and SCOP. In PVs, seven SCOP domains are identified altogether (Figure 1 and Figure 2).

To visualise the global relationship of PV protein structural domains to all other structural domains, “Galaxy of folds” toolkit was used (see Materials and Methods). This “structure space” was generated by Alva et al. to study the homologous origin of SCOP SF and FOLDs (FOLD is another SCOP level; SFs are assigned to FOLDs) [34]. Three PV domains, L1, E2 transactivation domain (TAD) and E1 DBD are located on the sparse periphery of “Galaxy of folds” space where the repulsive forces (i.e., dissimilarity) is dominant over the attractive force (i.e., similarity). E2 DBD and E7 are located at intermediate positions (still a sparse region) and E6 is located in a dense region. E1 helicase domain is located in a very dense region containing many different P-loop ATPases (including other hexameric helicases). Therefore, only the E1 helicase domain has a significant evolutionary relationship to other known structural domains.

### 3.4. Structural Domains Found in PV Proteins According to SUPERFAMILY Analysis

“Galaxy of Folds” is based on solved structures. Thus, the apparent loss of connections with other structures might be because the relatives are not yet structurally characterised or not yet in a database. To overcome (at least partially) this problem, we used data from SUPERFAMILY resource [29,37]. The SUPERFAMILY resource incorporates SCOP structural domain assignments (based on HMM models) at SF level to all annotated proteins in fully sequenced genomes [37,42]. If a protein with a similar structure to already solved structure is found in another fully sequenced organism, the SUPERFAMILY approach should recognise and classify it accordingly. Additionally, assignments to SFs are also applied to “NCBI viral genomes” and UniProt sequences. Hence, it is possible to evaluate the phylogenomic distribution of structural protein domains without the need of solved structures for each individual organism. The only drawback is that at least one representative structure for a protein domain must be solved. In SUPERFAMILY resource, 7 SCOP domains are found in PV sequences (Table 3 and Figure 1) (collectively named PV_SFs, i.e., SCOP superfamilies found in PVs). SCOP database has a hierarchical tree-structure—protein domains are classified into families and families are assigned to superfamilies, which, in turn, are classified to FOLDs (henceforth capitalised FOLD means SCOP hierarchical level) and then to classes. E2 TAD, E2 DBD, L1, E6, and E7 domains are classified into SFs that have only one family, thereby being the only representatives of the superfamily (Table 3). In addition, E7, E6, and E2 TAD have their own FOLD (i.e., 1 family per SF and 1 SF per FOLD) and thus, they do not have close structural relatives according to SCOP in the current database. L1 protein domain is a member of SF_88648, which together with 4 other viral capsid protein SFs, “Nucleoplasmin-like core domain” (SF_69203) and “PHM/PNGaseF” (SF_49742) form the FOLD called “Nucleoplasmin-like/VP (viral coat and capsid proteins)”. E2 DBD is a member of SF_54957 which has one family per SF and the respective SF belongs to the “Ferredoxin-like” FOLD together with 58 other SFs. However, we note that according to SCOP authors the SF level in SCOP is the highest level of confident homologous relationship (so, the SF belonging to the same FOLD might be or might not be evolutionarily related). E1 helicase domain belongs to the highly populated family “Extended AAA-ATPase domain”, which, together with 23 other families, forms SF_52540 (“P-loop containing nucleoside triphosphate hydrolases”). E1 DBD is a member of SF_55464 (“Origin of replication-binding domain, RBD-like”) and forms its own family. This SF also consists of four other families. Three of them are clearly virus related: polyomavirus Large-T DBD, geminiviral Rep protein DBD and parvoviral Rep protein nuclease domain. The fourth family is “Relaxase domain”, a domain with DNA nicking activity responsible for the conjugation of bacterial plasmids and bacterial DNA. Respective domain in parvoviral and geminiviral Rep proteins and Relaxase domain belongs to Rolling Circle Replication (RCR) proteins with endonuclease activity [43].

### 3.5. Phylogenetic Distribution of PV_SF Domains

To evaluate the evolutionary history and potential origin of PV structural domains we analysed the phylogenomic distribution of PV (structural) domains using the SUPERFAMILY resource. As shown in Table 3 (see Table S3) five domains (E2_TAD, E2_DBD, L1, E6, and E7) are not found in cellular “complete genomes”. Thus, these domains do not have confident homologs in completely sequenced cellular organisms, even including structure-based homology. From the seven SCOP domains, only domains from E1 protein are found in cellular genomes. SF_52540 representatives (E1 helicase domain and relatives, including all P-loop NTPases) are found in every cellular genome in the database and SF_55464 (E1 DBD domain and relatives) is found in 13 sequences of 8 eukaryotes (distinct NCBI taxonomy IDs) and in 261 sequences of 134 bacterial genomes (out of 1153 bacterial genomes in database) (Table 3, Table S3, Supplementary Materials, File S1). E1 DBD distant relatives are present in 5 fungi, 1 Alveolata, 1 Ameobozoa and 1 Viridiplantae and they are most likely relatives of Geminiviral Rep (Table 3, Supplementary Materials, File S1). The phylogenomic distribution of these hits is very sparse. Three out of five fungal hits are among Basidiomycota, but other 45 sequenced Basidiomycota in SUPERFAMILY database do not contain E1 DBD relatives (Figure S1).

To extend the search to non-complete genomes, UniProt sequences were used within SUPERFAMILY database. Additionally, HMM models of PV_SFs were run against all available databases using “hmmsearch”. This increased the number of hits of SF_55464 within the bacterial and eukaryotic sequences. For example, in eukaryotes, additional 23 species were found that coded potential SF_55464 homologs, increasing the number of Fungi species by 7 (including two close relatives of previously identified species), the number of Viridiplanate species by 11 (including 9 closely related *Dioscorea* species), two from Stramenopiles and two from Rhodophyta, and one in Rhizaria. Detailed analyses of the annotations of respective coding sequences show that in two Stramenopiles this domain is coded in mitochondrion and in *Rhodophyta* these sequences belong to algal plasmids (Supplementary Materials, File S1). The only domain, excluding E1, that seems to have a true positive hit in cellular organisms is E6 (SF_161229), which gives a hit to bacterium *Achromobacter xylosoxidans* AXX-A “Uncharacterised protein” F7T9H3_ALCXX (Table 3, Supplementary Materials, File S1). This sequence fits equally well into E6 structure and into ferredoxin structures according to LOMETS [32], a protein threading meta server which was used to verify sequence based homology predictions. Thus, only domains from the E1 protein show confident deeper evolutionary connection to cellular proteins.

Both domains found in cellular organisms (SF_52540 and SF_55464) are also found in other viruses (including all members of *Polyomaviridae*) and plasmids. In addition, representatives of SF_88648 (L1 protein) and SF_54957 (E2 DBD and relatives) are found only in viruses. Homologs of L1 protein (SF_88648) are found only in *Polyomaviridae*. E2 DBD relatives are found in a subset of gammaherpesviruses. In “NCBI viral genomes” the SF_55464 is also found in several *Parvoviridae, Geminiviridae*, in two *Betaherpesvirinae*, in one *Circoviridae* and *Siphoviridae* (relaxase domain); and in nine viruses recently classified as *Genomoviridae* (*Ge*—for geminivirus-like, *nomo*—for no movement protein) [44]. Among *Genomoviridae*, three sequences are classified into Gemycocircularvirus genus (Gemini-like myco-infecting circular virus) [44]. The members of SF_55464 (more precisely, mostly the relaxase domain) are also found in more than 400 bacterial plasmid sequences and notably, only in a single bacterial virus. In plasmid subset of SUPERFAMILY sequences, the SF_55464 is also found in one eukaryotic plasmid pPT4-NU with red algal host *Pyropia tenera* (this sequence was found also in SUPERFAMILY UniProt sequences and in HMMER search). In addition to different viral families and very few eukaryotes, E1 DBD connects PVs confidently with bacteria and bacterial plasmids.

### 3.6. Occurence of PV Protein Domains in Three Superkingdoms

**Figure 3:**
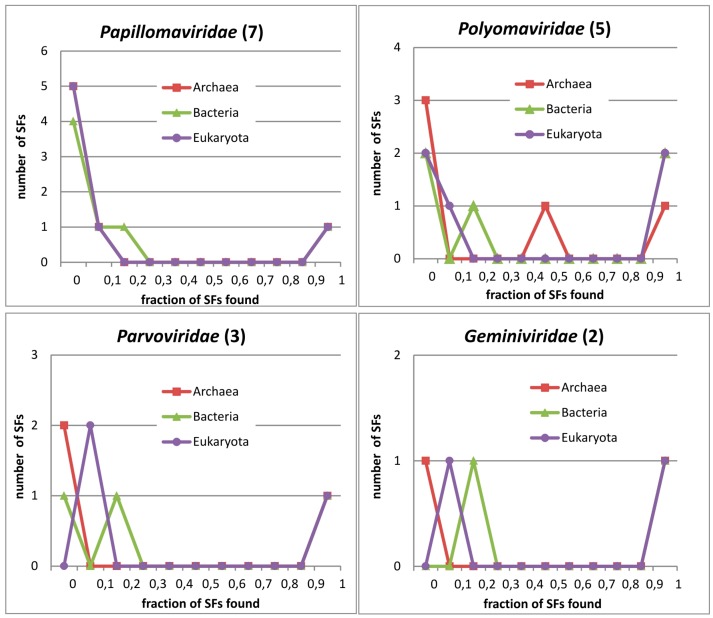
Distribution of protein domains in viral families by superkingdoms. Each figure shows data for the corresponding viral family. The number in the parentheses on titles corresponds to the number of distinct domains (SF) found in the respective viral family. The *y*-axis shows the number of domains (SF) from the viral family, covered by any of the three superkingdoms. The *x*-axis shows the decile of the genomes where the viral protein domains are found by superkingdoms. In panel *Papillomaviridae*, the lines for Archaea and Eukaryota overlap.

As shown above, PVs do have a connection with other superkingdoms on some levels. To visualise the occurrence of PV domains (and other similar small viruses) in cellular superkingdoms, we generated Figure 3. This is based on raw data because performing controls similar to PV_SF subset to all of the viruses in the figure would be extremely time-consuming. Figure 3 shows how many protein domains in corresponding viral family are found in the genomes of cellular superkingdoms (shown in percentage). In general, bimodal distribution can be observed (more viral families are covered in the Figure S2), which means that the shared protein domains between viruses and superkingdoms can usually be found in a small percentage of the cellular genomes or in most of them. For example, PVs have one domain (SF_52540) which is found in almost all organisms (value 1 on *x*-axis on Figure 3, panel *Papillomaviridae*) and one domain (SF_55464) is found in more than 0% and less than 10% of Eukaryotic genomes in the database. The rest of the domains (SF_51332, SF_54957, SF_88648, SF_161229 and SF_161234) are not found in any Eukaryotes. Similarly, two domains (SF_161229 and SF_161234) are found in more than 0% and less than 10% of bacteria and one domain (SF_55464) is found in more than 10% and less than 20% of bacterial genomes (Figure 3 and Table S3). Potential PV relatives (*Polyomaviridae*, *Geminiviridae*, and *Parvoviridae*) have a similar bimodal distribution, with a high fraction of domains found only in viruses and very few in cellular genomes. Additionally, *Polyomaviridae* encodes chaperone DnaJ domain (SF_46565) which is also found in half of Archaea genomes.

Bimodal distribution is not specific for small DNA viruses. Large DNA viruses, like members of *Herpesviridae* (and *Gammaherpesvirinae*, sharing E2 DBD with PVs) also have a bimodal distribution. However, they have a much higher fraction of proteins found in almost every cellular organism (data shown in Figure S2). Collectively, RNA viruses have a higher fraction of domains (either SF domains or PfamA domains) found only in viruses [40]. However, several dsDNA viruses like PVs (point *x* = 0.7, *y* = 0.5 in Abroi 2015 Figure 3), *Polyomaviridae* (0.6; 0.4), *Herpesviridae* (0.8; 0.05) and *Adenoviridae* (0.65; 0.1), have a fraction of virosphere-specific domains (i.e., domains found exclusively in the virosphere) as high as RNA viruses have (Figure S3).

### 3.7. Phylogenomic Distribution of the E1 SF_55464:SF_52540 Domain Pair

The P-loop NTPase (SF_52540) domain is very abundant in nature and therefore not very informative without much deeper analyses. However, PV E1 protein contains SF_55464 and SF_52540 domain, forming a domain pair. Thus, we decided to examine whether this domain pair is found elsewhere in the biosphere. In the SUPERFAMILY version used, SF_55464 and SF_52540, if on the same protein, are always in the same order, SF_55464 N-terminal and SF_52540 C-terminal, agreeing with previous studies showing that convergent evolution of protein architectures is rare [42]. As expected, this combination is found in all PVs and in all polyomaviruses (when we exclude database misannotations) (Table 3 and Table 4). In *Parvoviridae* species, which have an annotated SF_55464 domain, SF_52540 is also present. This domain pair is also found in bacterial plasmids and in more than 100 bacterial species, but not in any Archaea and only in some eukaryotes (Table 3, Supplementary Materials, File S1). We note that databases often do not discriminate between bacterial chromosome and plasmid (sometimes there is no clear border between them either). Most (but not all) of the plasmid (and bacterial) sequences having SF_55464 also have SF_52540 (Table 4). Among the 20 plasmid sequences with a single SF_52540 (i.e., domain architecture similar to PV E1 protein), nine belong to phytoplasma (obligate bacterial parasites of plant phloem tissue) plasmids (Supplementary Materials, File S1). These nine phytoplasma plasmid sequences have domain organisation most similar to PVs. E1_DBD relatives (SF_55464) together with P-loop NTPase (SF_52540) are found in very few eukaryotes with very sparse phylogenomic distribution. In the SUPERFAMILY database, this combination is found in three Fungi (all in Basidiomycota), one Alveolata and one Amoebozoa. Excluding PVs, *Polyomaviridae* and *Parvoviridae*, this combination is found in 21 viruses (21 distinct NCBI taxonomy IDs) including 14 members of *Geminiviridae*. From seven remaining sequences in the “NCBI viral genomes” dataset with both SF_55464 and SF_52540, three belong to *Genomoviridae*, two to *Herpesviridae* and one each to *Circoviridae* and *Siphoviridae* (Supplementary Materials, File S1). Thus, according to SUPERFAMILY data the PV replicative helicase has evolutionary connections with *Polyomaviridae* and *Parvoviridae,* as well as deeper connections with *Geminiviridae,* bacterial conjugative plasmids, including phytoplasma plasmids, and with bacteria.

## 4. Discussion

Several aspects of the molecular biology of PVs are quite well known, however, the origin and the evolutionary relationship to other organisms is still enigmatic. In this work, the occurrence of PV protein domains was used to study the relations of PV domains with other domains characterised so far and to study the origin and/or evolution of PV proteins and PVs.

**Figure 4:**
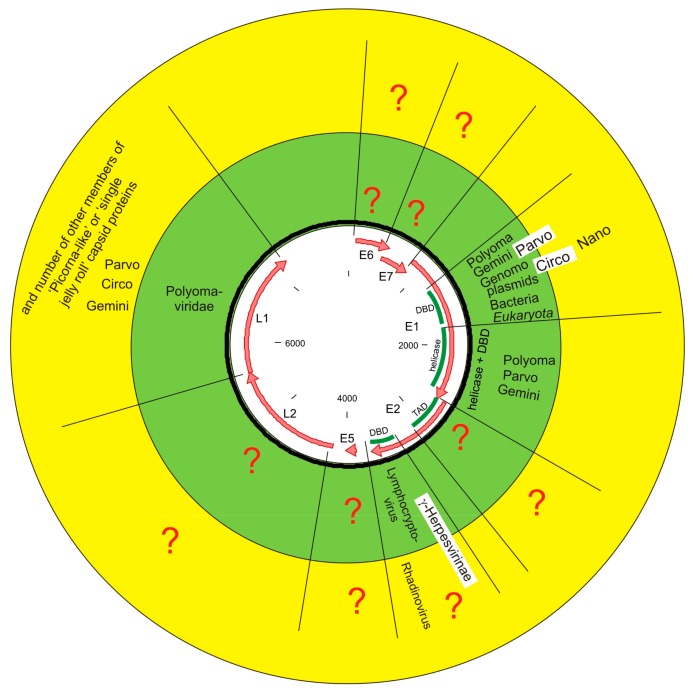
Summary figure of the relationship of PV domains with other parts of the biosphere. Virus family names are abbreviated without “-*viridae*” suffix. In the green circle, the relationships according to SCOP and SUPERFAMILY resource are shown. In the yellow circle, the relationships according to extended structural analysis from published articles and structures are shown. Genera *Lymphocryptovirus* and *Rhadinovirus* are subfamilies of *γ-Herpesvirinae.* For E1 helicase domain only evolutionary relationship via domain pair SF_55464:SF_52540 are shown.

PVs, similar to several other viral families, encode proteins without detectable structural homologs in cellular organisms [45]. This trend can be quantitatively evaluated in different ways [40]. As shown in Figure 3 (see also Figure S3) and in the analysis of protein domain occurrence at higher taxonomic levels in citation [40], PVs have a high fraction of protein domains not found in cellular superkingdoms or are found in a small fraction of cellular genomes. In this aspect (location of PV in Figure S3 and shape of the PV lines in Figure 3 and Figure S2 compared to Figure 4 in Reference [40]), PVs and *Polyomaviridae* are more similar to RNA viruses and ssDNA viruses than dsDNA viruses. That kind of bimodal or U-shape distribution is confirmed also independently at the structural level. Relationship of PV protein structural domains to other structural domains was assessed and visualised with “Galaxy of folds” toolkit. Only E1 helicase domain locates at a densely populated region (close relationship) and at least four domains locate at very sparse regions (Figure 2).

### 4.1. SUPERFAMILY Limitations

The SUPERFAMILY resource is a useful tool for deep evolutionary studies; unfortunately, it has its own limitations. Different HMM models of SCOP families from the same SF may not recognise easily the sequences from (structural) sibling family, especially in the case of viruses. For example, when using HMM model of PV E1 DBD domain and searching it against all the known sequences, it does not recognise Large-T antigen DBD sequences from *Polyomaviridae*. However, PV E1 DBD and *Polyomaviridae* Large-T antigen DBD are classified to the same SF in SCOP. In SUPERFAMILY, the SF hits are collected as a union of all of the respective SF HMM results [37]. In addition, SUPERFAMILY is limited to protein structural domains classified in SCOP. Unfortunately, not all protein structures of interest are in the SCOP database (not in SCOP 1.75 [35], SCOP2 [46] or SCOPe [47]).

Because of the gap between structural classification and current data in PDB database, biologically/virologically suspicious results were re-evaluated using most recent data. For example, SF_52540 was found in most *Parvoviridae* species but SF_55464 only in a subset. The structure of respective domain in SCOP is solved for Adeno-associated virus (*Dependoparvovirus*, *Parvovirinae*) (SCOP and PDB representative “1m55”). The HMM model based on “1m55” recognises protoparvoviruses (*Parvovirinae*) but not bocaparvoviruses (*Parvovirinae*) on HMMER “hmmsearch”. However, based on published structures the structural and functional similarity of bocaparvovirus “4kw3”, dependoparvovirus “1m55” and protoparvovirus “4pp4” gives evidence that bocaparvoviruses and probably *Densovirinae* (another subfamily of *Parvoviridae* family) have a homologous domain to SF_55464 [48]. Hopefully, the next release of SCOP (and SUPERFAMILY) will include up to date viral structural information. To avoid our subjective bias, SUPERFAMILY data and extended structural analysis data were interpreted separately. The quality of the data in databases in this kind of studies is very important. The amount of data used in our work is still comprehensible, allowing us to test correctness/quality of input data and our results, however, in larger-scale analyses it is not feasible or indeed possible. One PV related example is the network-like relationship studies of dsDNA viruses [49]. In the publication, data showed a connection (Figure 1 in [49]) between PV and polyomaviruses, which corresponds most likely to Bandicoot Papillomatosis virus; a chimera, containing capsid proteins from PV and a replication protein with a DnaJ domain from polyomaviruses. This connection was misinterpreted by the authors in the text. Therefore, to avoid or minimise misinterpretations in large scale studies, each scientific society should keep the data as correct as possible, to give confidence to large-scale analysis results.

### 4.2. Capsid Protein Connects PVs with a Rest of the Virosphere

The PV major capsid protein L1 has structural relatives at the SF level only in *Polyomaviridae*. In addition to L1, *Polyomaviridae* also codes domains structurally similar to E1 DBD and E1 helicase (including hexamerisation subdomain). PVs and *Polyomaviridae* are the only known viruses with nucleosomes inside virion [50,51]. The thirty-year-old statement by Favre et al. “The existence of a viral core containing DNA and cellular histones may be a further common structural characteristic of papovaviruses.” (Papovaviruses—old name of PVs and polyomaviruses together) is still valid and this characteristic is not only common but also specific for these viruses [50]. Thus, there are several lines of evidence that PVs and *Polyomaviridae* are clearly evolutionary related.

According to published non-hierarchical structural analysis and supported by the common FOLD level in SCOP, PV L1 and *Polyomaviridae* major capsid protein VP1 belong to the “single jelly-roll” (eight-stranded beta barrel) capsid lineage also called “Picorna-like lineage”. The single jelly-roll capsid lineage contains capsid proteins from a number of other viral families, including *Circoviridae*, *Geminiviridae*, and *Parvoviridae* together with numerous families of RNA viruses [52,53]. Viral families in this lineage have different replication strategies and have host ranges both from Eukaryota and Bacteria. As noted earlier, SCOP classification to the same FOLD level does not guarantee a common ancestor, however, it also does not exclude it. Thus, most likely PVs are connected to the wider virosphere via their major capsid protein L1.

### 4.3. E2 DBD Most Likely Does Not Originate from Gammaherpesviruses

As summarized in Figure 4, E2 DBD domain has connection only with gammaherpesviruses. According to SUPERFAMILY results and HMMER searches only members of genus *Lymphocryptoviruses* gives significant hits. However, published structures of rhadinovirus (genus *Rhadinovirus* is another member of *Gammaherpesvirinae* subfamily) proteins (PDB codes 4blg, 2yq1, 4k2j and 5a76) prove that functionally and structurally homologous proteins are found also in rhadinoviruses [54,55,56,57]. The divergence time of gammaherpesviruses, where the SF_54957 domain is found have not been estimated explicitly; however it is possible to estimate their potential divergence time to no more than ~200 million years ago from published data [58,59]. PVs have existed at least ~315 million years [23] and assuming virus-host co-divergence also for fish viruses, the PVs are most likely more ancestral, at least ~415 million years [60]. Therefore, PV E2 DBD does not originate from gammaherpesviruses, at least not after their divergence.

### 4.4. Replication Protein Connects PVs with a Rest of Biosphere

SF_55464 is also found in more than hundred bacterial species (Table 3) with wide and sparse phylogenomic distribution. Most of the bacterial hits have best *e*-value for the relaxase HMM model (as in the case of bacterial plasmids). Non-relaxase hits in Bacteria are found only in 5 phytoplasma species. Extending to noncomplete genomes increases the number of bacterial hits to a few thousands of sequences. Thus, at least via the relationship with the relaxase domain, PVs have connections with bacteria and bacterial plasmids. The relationship of geminiviral replication proteins to plasmid have been published, however the direction of the transfer is not clear [61,62].

Phylogenetically closest genomes to currently known PV hosts (Vertebrates) where SF_55464 is found in SUPERFAMILY database are among Fungi (Supplementary Materials, File S1). Extending the search to non-complete genomes we also found SF_55464 in some Metazoa, however, closer examination shows, that they are all most likely misannotations. These sequences were almost identical to Bacterial ones and, if we exclude very recent “from Bacteria to Eukaryota” transfer (which is possible but very unlikely), these sequences are most likely contaminants or a part of the sequenced organism’s microbiota. Detailed examination of true positive eukaryotic hits identified that in Stramenopiles this domain is coded by mitochondrial DNA, widening the phylogenetic distribution of RCR domains. All SF_55464 true positives in eukaryotes give best hit to geminiviral Rep HMM model. To test whether the eukaryotic hits are taxonomically restricted sequences or just a moderately divergent member of some other protein domain family we performed reciprocal sequence search using SF_55464 eukaryotic hits (parts of sequences corresponding to SF_55464) as a query in “phmmer” and “tblastn”. Only sequences from three organisms belonging to *Basidiomycota* (*Serpula lacrymans var. lacrymans* S7.9, *Pisolithus tinctorius* Marx 270 v1.0 and *Laccaria bicolor* S238N-H82) recognised each other SF_55464 sequences (and after them viral sequences from *Geminiviridae* and *Genomoviridae*). All other eukaryotic sequences give hits only to viral sequences mostly from *Geminiviridae* and *Genomoviridae*. Thus, in the sequenced biosphere, these eukaryotic hits do not have close homologs in other organisms (even those not yet annotated as protein). This indicates that these sequences are taxonomically restricted and not on the periphery on some unidentified protein domain family. Therefore, PVs have connections to the sequenced eukaryotic world only via distant relatives in the virosphere.

SF_55464 is found in all or almost all members of PV, *Polyomaviridae*, and *Geminiviridae*. It is also found in 10 viruses currently classified into the new proposed family *Genomoviridae* and in a single member of *Circoviridae* (out of 45 in the database). In the phylogenetic tree of Rep proteins of circular single-stranded DNA (ssDNA) viruses, genomoviral Rep proteins form a well-supported monophyletic clade which branches as a sister group of *Geminiviridae* and they both are more distantly related to *Circo*- and *Nanoviridae* [44]. The Rep protein tree is supported by structural analyses showing that Rep proteins of *Geminiviridae*, *Circoviridae* and *Nanoviridae* are indeed structurally related [44,48]. Nanovirus and circovirus Rep protein structures are not yet classified in SCOP. Thus, in the virosphere, extended structural analyses of E1 DBD relatives connect PV with *Polyomaviridae*, *Parvoviridae*, *Geminiviridae*, *Circoviridae*, *Nanoviridae*, and *Genomoviridae*. However, connection outside the virosphere is still restricted to bacterial plasmids, bacteria, very few eukaryotes and few red algal plasmids.

### 4.5. Closest Domain Pair of E1 Protein Is Found Far from Known PV Hosts

Since SF_55464 and SF_52540 coexist as a domain pair in E1 protein, the existence of this pair in other genomes was studied. In Bacteria, this combination is found in 119 species in SUPERFAMILY “complete genomes”, mostly in combination of “relaxase” domain with “Tandem AAA-ATPase domain”. This domain pair was present in very few eukaryotes with very sparse phylogenomic distribution in three Fungi, one Alveolata and one Amoebozoa. In contrast to Bacterial sequences they have best fit to geminiviral “DNA-binding domain of REP protein” and “Extended AAA-ATPase domain” HMM models.

In the case of viruses, E1 domain pair was found in *Polyomaviridae*, *Parvoviridae*, *Geminiviridae*, and in 21 other viruses. Sharing SF_55464 and SF_52540 (including the hexamerisation subdomain) is true for *Polyomaviridae* and *Parvoviridae* (or at least for *Parvovirinae*). PVs and *Polyomaviridae* are dsDNA viruses; however, *Parvoviridae* belongs to ssDNA viruses encapsulating linear ssDNA. Two proteins coded by human herpesviruses (HHV) from this list belong to roseloviruses, namely HHV6A and HHV6B. They encode a protein with most likely parvovirus origin. Herpesviruses are known helperviruses for some parvoviruses [63,64] and according to phylogenetic distribution of this protein (and phylogenetic tree) there have been from virus-to virus transfer with direction from parvoviruses to roseloviruses (during the last 100 MYA as estimated from [58,59]).

In plasmids, SF_55464 and SF_52540 domain combination is also found (Table 4). In most of the plasmids, the sequence regions assigned to SF_55464 have the best *e*-value for “relaxase” HMM model and regions assigned to SF_52540 have the best hit to “Tandem AAA-ATPase” HMM (like in Bacteria). Only the sequences of the phytoplasma plasmids and red algal plasmids of *Porphyra pulchra* have best *e*-value for geminiviral “DNA-binding domain of REP protein” HMM and SF_52540 models other than “Tandem AAA-ATPase”.

Considering “Virus to host” and “Host to virus” gene transfers and recombination of different viruses as well as accepting the statement by Rohwer and Barott “When considering the virosphere, extremely unlikely events become probabilistic certainties.” it is very difficult to estimate the evolutionary history or trajectory of these domains [65]. It is possible to generate the phylogenetic tree of these sequences but it is much harder to find a root. Work on the age of some of these viral genera and families may give some information and restrictions, but this is beyond the scope of the current study.

## 5. Conclusions

Summarizing over all protein domains of PV, only domains coding less than half of total annotated coding sequences show confident evolutionary connection to the rest of biosphere. This half include ~1/5 of total amino acids (E1 DBD and helicase) showing connection to sequenced and annotated cellular proteins and less than 1/20 of total amino acids (E2 DBD) showing connection with gammaherpesviruses.

PVs are clearly related to *Polyomaviridae*, sharing structural homologs of capsid protein and two domains of replication protein at SCOP SF level. Both viral families have dsDNA viral genomes packed into nucleosomes inside the viral particle. *Parvoviridae* shares two replication related domains and, including extended structural similarity, also the capsid protein with PVs and with *Polyomaviridae*. As ssDNA viruses, *Parvoviridae* do not have nucleosomes in virions.

The relationship of PV, *Polyomaviridae* and *Parvoviridae* to *Geminiviridae*, *Circoviridae*, *Nanoviridae*, and *Genomoviridae* is not as clear and their exact relationship is out of the scope of this work. They all have SF_55464 and according to extended structural analysis, they all (except *Nano*- and *Genomoviridae*) have common capsid protein (there are no structural predictions for *Nano*- and *Genomoviridae* capsid protein).

The major capsid protein L1 and replication protein E1 connect PVs to the rest of the virosphere, E1 DBD also connects PVs to bacterial plasmids, bacteria and red algal plasmids. Excluding the E1 helicase domain, the connections to eukaryotic protein domains are almost non-existent, even including available structural information. There are clear connections with other parts of the biosphere but the exact evolutionary trajectory of PV proteins is not yet known. There are still almost no hints as to how PVs as a whole/entirety originate and how they become vertebrate and epithelium specific. The evolutionary history of other PV protein domains, which have not been found in cellular organisms, is as mysterious.

Most likely the last common ancestor of PV, *Polyomaviridae*, and *Parvoviridae*, or more precisely, the genome coding the ancestral replication and/or capsid protein of these viruses, inhabited a marine environment. Only very few non-fungal and non-vertebrate marine eukaryotic genomes are sequenced. Thus, most likely, we have an unexplored sequence and structure space in both cellular and viral taxons, as well as in other types of mobile elements in marine environments. Further characterisation (sequencing is only one part of the characterisations) of this and other biotopes will give more information and thus more hints on the origin of PV proteins. On the other hand, some connections between PVs and other viruses or cellular organisms may be lost forever due to gene loss events. For example, in the PV family, the E6 gene was lost at least twice in different virus clades [15].

To our current knowledge, PVs are connected to the rest of biosphere via replication and major capsid proteins. The origin and/or evolutionary history of other domains are still unknown. This makes the question “When and how did PV originate?” of continuing interest.

## Figures and Tables

**Table 1:** Domain coverage comparison in UniProt “complete proteomes” in PfamA and SUPERFAMILY database.

	PfamA_28 *			SUPERFAMILY		
	Sequence Coverage ^1^	Residue Coverage ^2^	No. of Genomes	Sequence Coverage ^1^	Residue Coverage ^2^	No. of Genomes
Archaea	73.8	58.0	182	64.4	61.1	122
Bacteria	82.0	63.3	3513	67.6	62.6	1153
Eukaryota	67.9	38.6	422	56.9	38.8	440
Viruses	84.4	65.7	1198	34.3	28.1	4041
dsDNA viruses	62.5	52.9	270	24.8	25.4	1758
*Papillomaviridae*	90.8	83.8	76	69.5	57.5	125
*Polyomaviridae*	92.5	70.3	10	60.2	65.3	50
*Parvoviridae*	74.7	56.3	23	69.5	55.0	81
*Geminiviridae*	97.0	79.9	34	18.5	15.1	332
*Herpesviridae*	74.2	53.6	28	27.6	20.7	57

^1^ Sequence coverage shows the percentage of proteins in a genome which are covered by at least one domain. ^2^ Residue coverage shows the percentage of amino acids from all proteins of a genome which are within domain models. * PfamA_28 data from “complete genomes” subset.

**Table 2:** PV_PfamA domain occurrence in biosphere.

		*Papillomaviridae* ^1,5^	PDB PfamA_28 ^2^	PfamA Domain Length ^3^	PDB PfamA_31 ^2^	Best Coverage of PfamA by PDB (% aa)	Eukaryota (Proteomes) ^1^	Bacteria (Proteomes) ^1^	Archaea (Proteomes) ^1^	Viruses ^1,4^	Eukaryota (Full up) ^1^	Bacteria (Full up) ^1^	Archaea (Full up) ^1^	Viruses (Full up) ^1,4,6^	HMMER E ^1^	HMMER B ^1^	HMMER A ^1^	HMMER V ^1,6^
PF00500	Late_protein_L1	76	10	498	18	0.96	-	-	-	-	-	-	-	-	-	-	-	-
PF00508	PPV_E2_N	76	8	200	8	0.98	-	-	-	-	-	-	-	-	-	-	-	-
PF00511	PPV_E2_C	76	16	80	16	0.96	-	-	-	-	-	-	-	-	-	-	-	-
PF00513	Late_protein_L2	76	0	525	0		-	-	-	-	-	-	-	-	-	-	-	-
PF00518	E6	71	7	110	8	0.99	-	-	-	-	-	-	-	-	-	-	-	-
PF00519	PPV_E1_C	74	7	432	8	0.96	-	1	-	-	-	20	-	1	-	1	-	1
PF00524	PPV_E1_N	72	0	121	0		2	-	-	-	4	-	-	-	-	-	-	-
PF00527	E7	71	3	93	4	0.50	-	-	-	-	-	-	-	-	-	-	-	-
PF02711	Pap_E4	25	0	95	0		-	-	-	-	-	-	-	-	-	-	-	-
PF03025	Papilloma_E5	9	0	72	0		-	-	-	-	-	-	-	-	-	-	-	-
PF05776	Papilloma_E5A	5	0	91	0		-	-	-	-	-	-	-	-	-	-	-	-
PF08135	EPV_E5	3	0	43	0		-	-	-	-	-	-	-	-	-	-	-	-

“-” No true positive hits were found. ^1^ Number of distinct proteomes/species in database with given taxonomic restrictions coding respective domain. ^2^ Number of Protein Data Bank (PDB) entries for respective PfamA domain. ^3^ Model length. ^4^ Excluding papillomaviruses. ^5^ 76 PV proteomes in this database. ^6^ Excluding *Polyomaviridae* and *Parvoviridae*.

**Table 3:** PV_SF domain occurrence in biosphere.

SCOP/SF ID	Classification	SF/FOLD	Families/SF	Description	PV	Viruses ^1^	Plasmids ^2^	Archaea	Bacteria	Eukaryota	HMMER A	HMMER B	HMMER E	HMMER V ^1^
55464	d.89.1	1	5	Origin of replication-binding domain, RBD-like (E1 DBD)	123	424/**15** *	420	-	134	**8**	-	4038	**32**	1563/169 *
52540	c.37.1	1	24	P-loop containing nucleoside triphosphate hydrolases (E1 helicase)	123	2346	19971	122	1153	440	ND	ND	ND	ND
51332	b.91.1	1	1	E2 regulatory, transactivation domain (E2 TAD)	123	-	-	-	-	-	-	-	-	-
54957	d.58.8	59	1	Viral DNA-binding domain (E2 DBD)	123	**4**	-	-	-	-	-	-	-	**6**
88648	b.121.6	7	1	Group I dsDNA viruses (L1)	123	**50**/- *	-	-	-	-	-	-	-	170/- *
161229	g.90.1	1	1	E6 C-terminal domain-like	115	-	-	-	-	-	-	**1?**	-	-
161234	g.91.1	1	1	E7 C-terminal domain-like	108	-	-	-	-	-	-	-	-	-
55464:52540				DBD + helicase	123	**7**	356	-	119	**5**	-	ND	**10**	

“-” No true positive hits were found. “ND” Not determined. “Underlined” Number of primary hits. “Bold” Number of true positive hits. * Number of true positives without *Polyomaviridae*, *Parvoviridae* and *Geminiviridae.* “?” Questionable result. ^1^ Excluding papillomaviruses. ^2^ Number of proteins. Non-redundant set of genomes contain 122 Archaeal, 1153 Bacterial, and 440 Eukaryotic species) (i.e., redundant strains and isolates removed). DBD: DNA-binding domain; TAD: transactivation domain. For more detailed information, see Table S3.

**Table 4:** Number of sequences containing SF_55464 with different domain architectures.

No. of 52540 Domains	PV ^1^ 123 *	*Polyomaviridae* ^2^ 50 *	*Parvoviridae* 81 *	*Geminiviridae* ^3^ 332 *	Other Viruses	Plasmids	Bacteria	Eukaryota
0	1	0	0	350	10	64	35	4
1	122	49	33	14	6	20	20	5
2	0	0	0	0	1	334	183	0
3	0	0	0	0	0	2	1	0

^1^ In HPV53, only DBD part of E1 is annotated. ^2^ *Polyomaviridae* Merkel cell polyomavirus does not have annotated full-length Large-T protein in this version of the database used (in current version of NCBI viral genomes it already has). ^3^ Geminiviruses have often more than one replication protein isoform annotated. * Number of genomes in the respective viral family.

## Supplementary Materials

The following are available online at www.mdpi.com/1999-4915/9/9/240/s1.
