# Supplementary material for: The Enigmatic Origin of Papillomavirus Protein Domains
Source: Viruses. 2017 Aug 23;9(9):240. doi: 10.3390/v9090240 (PMC5618006; doi:10.3390/v9090240)
Supplement: Supplementary file 1 [file viruses-09-00240-s001.zip › Supplementary_Data.pdf]

viruses

MDPI

Supplementary

Table S1. PV_PfamA domain occurrence in biosphere.

		Papillomaviridae [1,5]	**PDB PfamA_28** [2]	**PfamA domain length** [3]	**PDB PfamA_31** [2]	**Best coverage of PfamA by PDB (% aa)**	**Eukaryota** (proteomes) [1]	**Bacteria** (proteomes) [1]	**Archaea** (proteomes)	**VIRUSES** (proteomes) [1,4]	**Eukaryota** (full UP) [1]	**Bacteria** (full UP) [1]	**Archaea** (full UP)	**VIRUSES** (full UP) [4]	**HMMER E**	**HMMER B**	**HMMER A**	**HMMER V**
PF00500	Late_protein_L1	76	10	498	18	0.96	0	0	0	0	1/0	0	0	0	9/0	2/0	0	0
PF00508	PPV_E2_N	76	8	200	8	0.98	0	0	0	0	0	0	0	0	7/0	24/0	0	0
PF00511	PPV_E2_C	76	16	80	16	0.96	0	0	0	0	0	0	0	0	9/0	2/0	0	0
PF00513	Late_protein_L2	76	0	525	0		0	0	0	0	1/0	0	0	0	8/0	1/0	0	0
PF00518	E6	71	7	110	8	0.99	1/0	1/0	0	0	2/0	1/0	0	0	7/0	1/0	0	0
PF00519	PPV_E1_C	74	7	432	8	0.96	6/0	2/**1**	0	0	19/0	28/**21**	0	242/ ND/**1**	35/0	2/**1**	0	822/ ND/**1**
PF00524	PPV_E1_N	72	0	121	0		2/**2**	0	0	0	4/**4**	0	0	0	5/0	3/0	0	0
PF00527	E7	71	3	93	4	0.50	0	0	0	0	1/0	1/0	0	0	4/0	2/0	0	0
PF02711	Pap_E4	25	0	95	0		0	0	0	0	0	0	0	0	0	1/0	0	0
PF03025	Papilloma_E5	9	0	72	0		0	0	0	0	1/0	0	0	0	1/0	1/0	0	0
PF05776	Papilloma_E5A	5	0	91	0		0	0	0	0	0	0	0	0	0	1/0	0	0
PF08135	EPV_E5	3	0	43	0		0	0	0	0	0	0	0	0	0	0	0	0

[1] Number of distinct proteomes/species in database with given taxonomic restrictions coding respective domain. [2] Number of PDB entries for respective PfamA domain. [3] Model length. [4] Excluding papillomaviruses. [5] 76 PV proteomes in this database. * Number before the slash shows all hits (distinct proteomes / species). Number after the first slash shows hits after removal of false positives as described in Materials and Methods. For viruses, after the second slash are the true positive hits without *Polyomaviridae* and *Parvoviridae*. "Bold" Number of true positive hits described in detail in Supplementary Materials, File S1.

Table S2. HMM model version used as query profiles in 'hmmsearch'

a) PfamA models

	**PfamA domain**	**HMM version number**
PF00500	Late_protein_L1	PF00500.17
PF00508	PPV_E2_N	PF00508.16
PF00511	PPV_E2_C	PF00511.16
PF00513	Late_protein_L2	PF00513.17
PF00518	E6	PF00518.16
PF00519	PPV_E1_C	PF00519.16
PF00524	PPV_E1_N	PF00524.17
PF00527	E7	PF00527.17
PF02711	Pap_E4	PF02711.13
PF03025	Papilloma_E5	PF03025.13
PF05776	Papilloma_E5A	PF05776.11
PF08135	EPV_E5	PF08135.10

b) SUPERFAMILY models

SF	HMM model ID	No. of sequences	Model Build date	Seed sequence (SCOP domain name)	SCOP family of seed	Viral family of seed sequence
55464	0052766	293	2008-09-10	d2fufa1	The origin DNA-binding domain of SV40 T-antigen	*Polyomaviridae*
55464	0043184	110	2005-09-08	d1r9wa_	Replication initiation protein E1	*Papillomaviridae*
55464	0037306	110	2005-09-08	d1f08a_	Replication initiation protein E1	*Papillomaviridae*
55464	0040790	22	2005-09-08	d1m55a_	Replication protein Rep, nuclease domain	***Parvoviridae***
55464	0040363	209	2005-09-08	d1l2ma_	DNA-binding domain of REP protein	***Geminiviridae***
55464	0042148	63	2005-09-08	d1p4da_	Relaxase domain	
55464	0041922	64	2005-09-08	d1omha_	Relaxase domain	
51332	0044126	120	2005-09-08	d1tueb_	E2 regulatory, transactivation domain	*Papillomaviridae*
51332	0036595	120	2005-09-08	d1dtoa_	E2 regulatory, transactivation domain	*Papillomaviridae*
51332	0043150	120	2005-09-08	d1r6na_	E2 regulatory, transactivation domain	*Papillomaviridae*
51332	0042936	119	2005-09-08	d1qqha_	E2 regulatory, transactivation domain	*Papillomaviridae*

54957	0045729	253	2008-09-10	d1a7ge_	Viral DNA-binding domain	*Papillomaviridae*
54957	0046292	253	2008-09-10	d1dbda_	Viral DNA-binding domain	*Papillomaviridae*
54957	0049130	253	2008-09-10	d1r8ha_	Viral DNA-binding domain	*Papillomaviridae*
54957	0035341	8	2005-09-08	d1b3ta_	Viral DNA-binding domain	***Herpesviridae***
54957	0046040	251	2008-09-10	d1by9a_	Viral DNA-binding domain	*Papillomaviridae*
54957	0046732	253	2008-09-10	d1f9fa_	Viral DNA-binding domain	*Papillomaviridae*
88648	0043624	14	2005-09-08	d1sida_	Papovaviridae-like VP	***Polyomaviridae***
88648	0046429	1104	2008-09-10	d1dzla_	Papovaviridae-like VP	*Papillomaviridae*
88648	0043775	13	2005-09-08	d1sva1_	Papovaviridae-like VP	***Polyomaviridae***
161229	0053919	315	2010-05-31	d2fk4a1	E6 C-terminal domain-like	*Papillomaviridae*
161234	0053733	163	2010-05-31	d2b9da1	E7 C-terminal domain-like	*Papillomaviridae*
161234	0053897	160	2010-05-31	d2ewla1	E7 C-terminal domain-like	*Papillomaviridae*

Table S3. PV_SF domain occurrence in biosphere

**SCOP/SF ID**	**Classification**	**SF/FOLD**	**Families/SF**	**Description**	**PV**	**Viruses** [1,3]	**Plasmids** [2,3]	**Archaea** [3]	**Bacteria** [3]	**Eukaryota** [3]	**HMMER A**	**HMMER B**	**HMMER E**	**HMMER V** [1]
55464	d.89.1	1	5	Origin of replication-binding domain, RBD-like (E1 DBD)	123/ 123	424/ ND/**15**	420/ ND	0	134/ ND	8/ **8**	0	4038/ ND	52/ **32**	1563/ ND/169
52540	c.37.1	1	24	P-loop containing nucleoside triphosphate hydrolases (E1 helicase)	122/ 123	2346/ND	19971/ ND	122/ ND	1153/ ND	440/ ND	ND	ND	ND	ND
51332	b.91.1	1	1	E2 regulatory, transactivation domain (E2 TAD)	122/ 123	0	0	0	0	0	0	25/0	7/0	0
54957	d.58.8	59	1	Viral DNA-binding domain (E2 DBD)	121/ 123	4/**4**	0	0	0	0	0	1/0	10/0	6/**6**
88648	b.121.6	7	1	Group I dsDNA viruses (L1)	125/ 123	50/ 50/0	0	0	0	0	0	2/0	7/0	170/ ND/0
161229	g.90.1	1	1	E6 C-terminal domain-like	115/ 115	0	0	1/0	7/0	0	0	1/**1**	9/0	0
161234	g.91.1	1	1	E7 C-terminal domain-like	108/ 108	0	0	0	2/0	0	0	2/0	4/0	0
SF_55464:SF_52540				DBD + helicase	122/ 123	106/ND/**7**	356	0	119	**5**	0	ND	**10**	

Number before the slash shows primary hits (number of genomes the SF is found). Number after the first slash shows true positives, after the second slash (in nonPV viral columns) number of true positives without *Polyomaviridae*, *Parvoviridae* and *Geminiviridae*.

[1] Excluding papillomaviruses. [2] Number of proteins. [3] SUPERFAMILY data from locally downloaded database of non-redundant set of genomes (including Archaea 122 genomes, Bacteria 1153 genomes, Eukaryota 440 genomes; i.e. redundant strains and isolates removed). "ND" Not determined. "Bold" Number of true positive hits described in detail in Supplementary Materials, File S1.

In the current version of SUPERFAMILY the sequences with assigned SF_55464 can be found in eukaryotes:
http://supfam.org/SUPERFAMILY/cgi-bin/allcombs.cgi?genome=euk;comb=55464;subdomain=y

in plasmids:
http://supfam.org/SUPERFAMILY/cgi-bin/allcombs.cgi?genome=pla;comb=55464;subdomain=y

and in viruses:
http://supfam.org/SUPERFAMILY/cgi-bin/allcombs.cgi?genome=vl;comb=55464;subdomain=y
* You can change the '55464' in URL to other SF numbers for respective data.

Domain composition of specified Uniprot sequences can be evaluated in
http://supfam.org/SUPERFAMILY/cgi-bin/gene.cgi?genome=up;seqid=U9TRZ4
* By changing Uniprot sequence names after last equation mark.

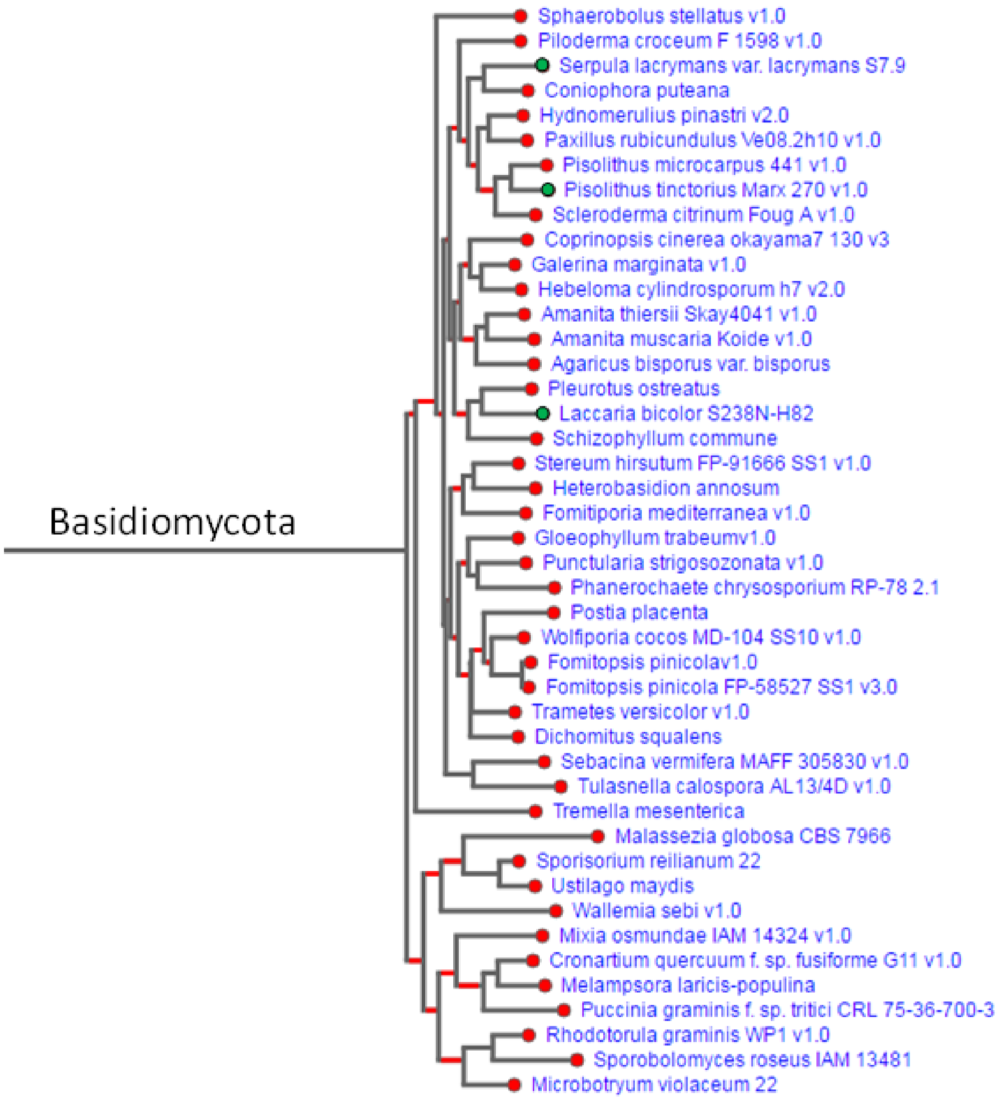

Figure S1. SF_55464 is found in three Basidomycota species (green circles). Species with red circle correspond to other Basidomycota proteomes in SUPERFAMILY. Tree from SUPERFAMILY resource according to method [1].

1. Fang, H.; Oates, M. E.; Pethica, R. B.; Greenwood, J. M.; Sardar, A. J.; Rackham, O. J. L.; Donoghue, P. C. J.; Stamatakis, A.; de Lima Morais, D. A.; Gough, J. A daily-updated tree of (sequenced) life as a reference for genome research. Sci. Rep. 2013, 3, doi:10.1038/srep02015.

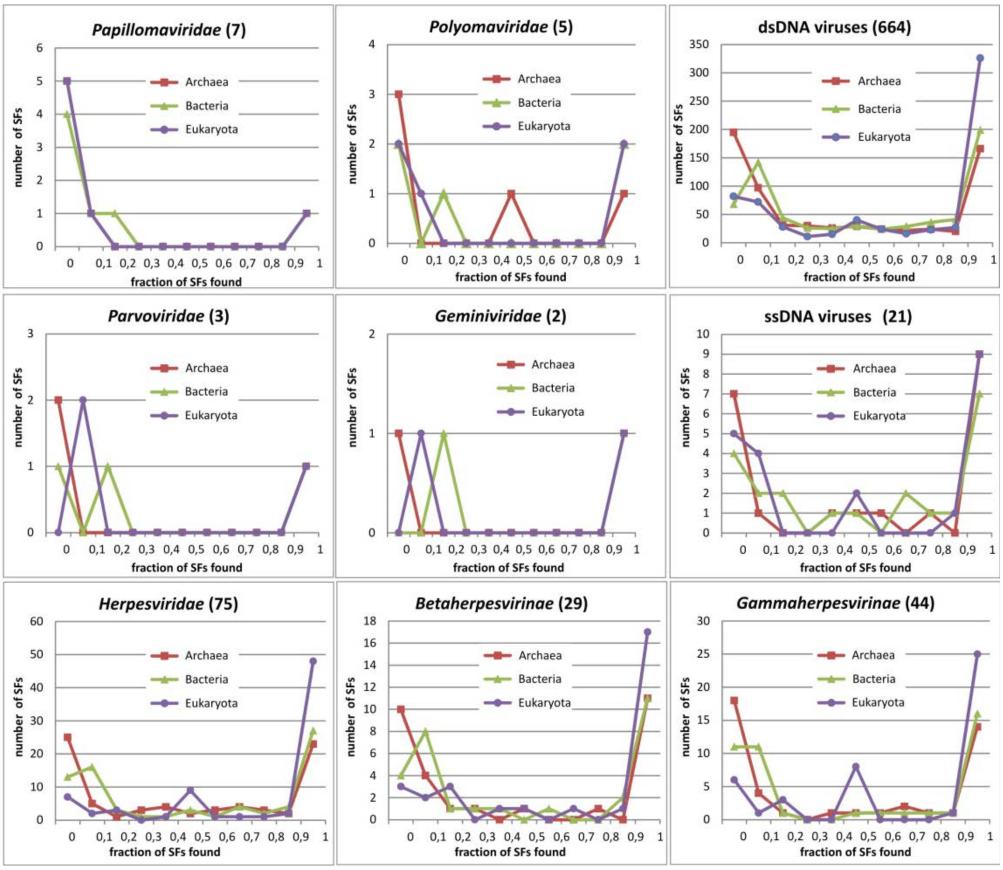

Figure S2. Distribution of protein domains in viral families by superkingdoms. Only data of the domains found in the viral family in the panel title are shown in each figure. The number in parentheses in the figure titles correspond to the number of distinct domains found in the respective viral family. On the x-axis, the decile of genomes by superkingdom where viral protein domains are found. The line shows the superkingdom for which the fraction is calculated. On the y-axis, the number of domains is given for those found in the taxon indicated in the title and the respective decile.

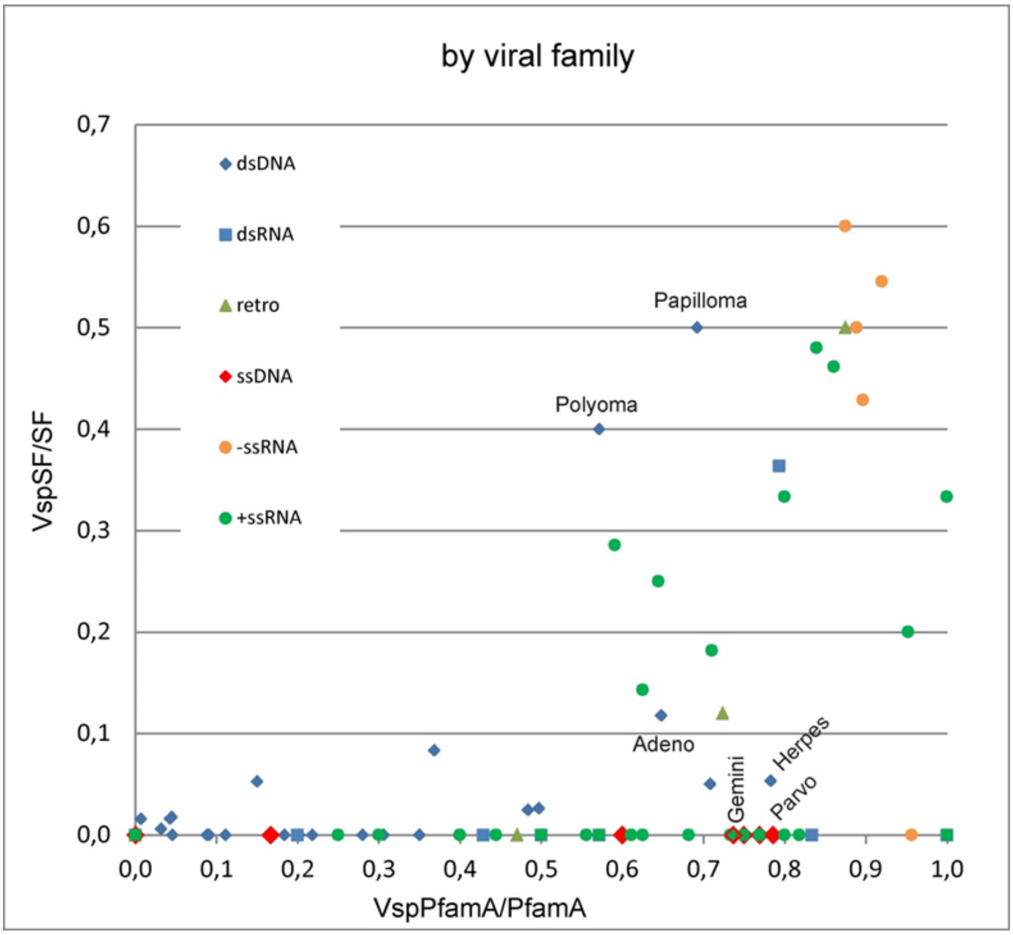

Figure S3. Ratio of virosphere-specific domains to total number of domains in that viral taxon. For respective taxons, the total number of assigned domains was calculated, as well as the number of virosphere-specific domains. The indicated ratios are shown on the axis. Every data-point corresponds to a viral family. Most of the viral family names have been omitted for clarity. For full names see citation [2] supplementary.

2. Abroi, A. A protein domain-based view of the virosphere-host relationship. *Biochimie* 2015, *119*, 231–243, doi:10.1016/j.biochi.2015.08.008.

Threading programs behind LOMETS metaserver.

Description of LOMETS on April 18th, 2017 from http://zhanglab.ccmb.med.umich.edu/LOMETS/ and https://zhanglab.ccmb.med.umich.edu/LOMETS/readme.txt.

LOMETS (Local Meta-Threading-Server) is an on-line web service for protein structure prediction [1]. It generates 3D models by collecting high-scoring target-to-template alignments from 9 locally-installed threading programs (FFAS-3D, HHsearch, MUSTER, pGenTHREADER, PPAS, PRC, PROSPECT2, SP3, and SPARKS-X).

Server(i)	Reference
1. cdPPAS	[7]
2. FFAS03	[8]
3. FFAS-3D	[11]
4. MUSTER	[2]
5. HHSEARCH	[3]
6. HHSEARCH-I	[3]
7. HHSEARCH-2	[3]
8. Neff-PPAS	[7]
9. pGenTHREADER	[10]
10. PRC	[9]
11. PROSPECT2	[6]
12. SPARKS-X	[4]
13. SP3	[5]
14. wdPPAS	[7]

[1] Wu, S.; Zhang, Y. LOMETS: A local meta-threading-server for protein structure prediction. *Nucleic Acids Res.* **2007,** *35, 3375-3382.*

[2] Wu, S; Zhang, Y. MUSTER: Improving protein sequence profile-profile alignments by using multiple sources of structure information. *Proteins* **2008,** *72, 547-556.*

[3] Soding, J. Protein homology detection by HMM-HMM comparison. *Bioinformatics* **2005,** *21, 951-960.*

[4] Zhou, H.; Zhou, Y. Single-body residue-level knowledge-based energy score combined with sequence-profile and secondary structure information for fold recognition. *Proteins,* **2004,** *55, 1005-1013.*

[5] Zhou, H.; Zhou, Y. Fold recognition by combining sequence profiles derived from evolution and from depth-dependent structural alignment of fragments. *Proteins,* **2005,** *58, 321-328.*

[6] Xu, Y.; Xu, D. Protein threading using PROSPECT: design and evaluation. *Proteins,* **2000,** *40, 343-354.*

[7] Yan, R.; Xu, D.; Yang, J.; Walker, S.; Zhang, Y. A comparative assessment and analysis of 20 representative sequence alignment methods for protein structure prediction. *Scientific Reports,* ***2013,*** *3: 2619.*

[8] Jaroszewski, L.; Rychlewski, L.; Li, Z.; Li, W.; Godzik, A. FFAS03: a server for profile--profile sequence alignments. *Nucleic Acids Res.* **2005,** *33(Web Server issue): W284-288.*

[9] Madera, M. Profile Comparer: a program for scoring and aligning profile hidden Markov models. *Bioinformatics.* **2008,** *24(22):2630-2631.*

[10] Lobley, A.; Sadowski, M. I.; Jones, D. T. pGenTHREADER and pDomTHREADER: new methods for improved protein fold recognition and superfamily discrimination. *Bioinformatics.* ***2009,*** *25(14):1761-1767.*

[11] Xu, D.; Jaroszewski, L.; Li, Z.; Godzik, A. FFAS-3D: improving fold recognition by including optimized structural features and template re-ranking. *Bioinformatics.* **2014,** *30(5): 660-667.*

Supplementary comments on using PfamA_28

There are several reasons why we used PfamA_28. PfamA_27 was released on March 2013 (based on Swiss 2012_06 + SP-TrEMBL 2012_06) and PfamA_28 was released on May 2015 (based on Swiss 2014_07 + SP-TrEMBL 2014_07). They are both based on full UniProt sequences. The next release, PfamA_29, is based on UniProtKB 'reference proteomes' (2015_08). In *Papillomaviridae* the 'reference proteomes' in PfamA_29 (15 species) do not have as good taxonomic coverage as 'complete proteomes' in PfamA_28 (76 species). Starting from PfamA_28 the seed sequences (that the HMM is based on) have started to move from UniProt sequences to UniProt 'reference proteome' sequences (**A, B**). If the reference proteomes cover most of the taxonomic diversity, then this change is very reasonable. Unfortunately, in some viral taxons the set of reference proteomes is far from real diversity. For example, in *Polyomaviridae* there is only one reference proteome in PfamA releases 29 to 31.

Papillomaviridae E1, *Polyomaviridae* Large-T and *Parvoviridae* NS1 most likely have a common ancestor. When the HMM model is built from a narrow set of sequences for one of them and as wide sequence set as possible for another, then the 'wide model' starts to recognise with high score the sequences that actually belongs to the other family. We see this phenomenon when searching relatives of E1 in full UniProt, where PF00519 recognises several polyomaviral and parvoviral sequences. In PfamA_29 the PF00519 also starts to recognise more sequences in *Polyomaviridae* and *Parvoviridae* as well as cellular organisms. This is due to the improper set of seed sequences (due to a biased set of reference proteomes) or to other model assignment parameters. Running a random set of these non-*Papillomaviridae* viral sequences against current PfamA profiles using 'hmmscan' in HMMER (www.hmmer.org) gives the best hits on NS1 (PF01057) or Large-T (PF06431) respectively.

On the next PfamA release this problem will most likely be addressed (at least for *Papillomaviridae* and *Polyomaviridae*) as there will be a representative set of reference proteomes for both viral families **(D).**

Footnotes on changes in Pfam A.

A) ftp://ftp.ebi.ac.uk/pub/databases/Pfam/releases/Pfam28.0/relnotes.txt

"As of release 28.0 we have begun to move our SEED alignments onto Reference Proteome sequences. In release 28.0 14190 families have SEEDs consisting of Reference Proteome sequences and 2040 have SEEDs consisting of non-Reference Proteome sequences.

We have now relaxed our policy on overlaps. From release 28.0 we now only check for overlaps within the Reference Proteome sequence set. We also now allow some overlaps at the ends of a match. We now allow overlaps which span less than 20% at either the N- or C-terminus of the lower scoring family where these overlaps occur in less than 1% of the family."

B) ftp://ftp.ebi.ac.uk/pub/databases/Pfam/releases/Pfam29.0/relnotes.txt
"As of Pfam 29.0, the sequence database that Pfam is based upon changed to UniProtKB **reference proteomes** (prior to that it was based on all of UniProtKB). A separate table for UniProtKB statistics is provided below for releases 29.0 onwards)."

"As of release 28.0 we started to move our SEED alignments onto Reference Proteome sequences. In release 29.0 12969 families have SEEDs consisting solely of Reference Proteome sequences. The rest of the families contain some SEED sequence(s) that are in UniProtKB 2015_08, but are not in UniProtKB reference proteomes 2015_08."

C) NAR 2016 database issue, https://doi.org/10.1093/nar/gkv1344
"We have applied two approaches to migrating *seed* alignments to the reference proteomes. The first method was to take the existing profile HMM and search against the reference proteome sequence database, align all significant matches (excluding partial matches) and make the resulting alignment 80% non-redundant. This alignment was used to construct a new profile HMM which was searched against UniProtKB. We compared the sensitivity of the new profile HMM with the results of running the original profile HMM against the same version of UniProtKB."

D) Number of complete or reference proteomes of selected viral families in different database releases:

Database and release	*Papilloma-viridae*	*Polyoma-viridae*	*Parvo-viridae*
PfamA_28 (complete proteomes)	76	10	23
PfamA_29 (reference proteomes)	15	1	11
PfamA_30 (reference proteomes)	37	1	11
PfamA_31 (reference proteomes)	37	1	12
Current UniProt 'reference proteomes' (May 2017)	166	50	15
